# Proteomic Analysis of Carbon Concentrating Chemolithotrophic Bacteria *Serratia* sp. for Sequestration of Carbon Dioxide

**DOI:** 10.1371/journal.pone.0091300

**Published:** 2014-03-11

**Authors:** Randhir K. Bharti, Shaili Srivastava, Indu Shekhar Thakur

**Affiliations:** School of Environmental Sciences, Jawaharlal Nehru University, New Delhi, India; Universidad Nacional de La Plata., Argentina

## Abstract

A chemolithotrophic bacterium enriched in the chemostat in presence of sodium bicarbonate as sole carbon source was identified as *Serratia* sp. by 16S rRNA sequencing. Carbon dioxide sequestering capacity of bacterium was detected by carbonic anhydrase enzyme and ribulose-1, 5- bisphosphate carboxylase/oxygenase (RuBisCO). The purified carbonic anhydrase showed molecular weight of 29 kDa. Molecular weight of RuBisCO was 550 kDa as determined by fast protein liquid chromatography (FPLC), however, sodium dodecyl sulphate polyacrylamide gel electrophoresis (SDS-PAGE) showed presence of two subunits whose molecular weights were 56 and 14 kDa. The Western blot analysis of the crude protein and purified sample cross reacted with RuBisCO large-subunit polypeptides antibodies showed strong band pattern at molecular weight around 56 kDa regions. Whole cell soluble proteins of *Serratia* sp. grown under autotrophic and heterotrophic conditions were resolved by two-dimensional gel electrophoresis and MALDI-TOF/MS for differential expression of proteins. In proteomic analysis of 63 protein spots, 48 spots were significantly up-regulated in the autotrophically grown cells; seven enzymes showed its utilization in autotrophic carbon fixation pathways and other metabolic activities of bacterium including lipid metabolisms indicated sequestration potency of carbon dioxide and production of biomaterials.

## Introduction

Carbon dioxide (CO_2_), one of the major green house gases (GHGs), whose concentration was stable at about 270 ppm has increased approximately by 38% to 380 ppm after industrial revolution. It is predicted that by the middle of this century, the concentration of CO_2_ will reach to 600 ppm, and by the end of the century it is likely to reach 700 ppm [1]. Increase in CO_2_ concentration may be mitigated by autotrophic and heterotrophic carbon fixation by plants and microorganisms. Microorganisms can acclimate to a wide range of carbon dioxide by carboxylating enzymes. Six different types of biochemical pathways viz. ribulose 1,5 biophosphate carboxylase/oxygenase (RuBisCO) in Calvin cycle, reductive citric acid cycle, reductive acetyl-coenzyme A pathway, 3-hydroxypropionate bicycle, hydroxypropionate-hydroxybutarate cycle and decarboxylate-hydroxybutyrate cycle, are reported to assimilate CO_2_ into biomass, and formation of cellular materials like biofuels and chemical products [2]. CO_2_ was present at relatively low concentrations in the atmosphere initially and often limiting substrate for photosynthetic carbon assimilation in plants and other photosynthetic and chemosynthetic microorganisms [3]. Autotrophic organisms have the ability to form cell material solely from inorganic carbon. This makes autotrophic processes a crucial component of the global carbon cycle. The balance between autotrophy and heterotrophy is a key factor regulating CO_2_ and O_2_ concentrations in the atmosphere, and it also affects the overall redox balance of the Earth [4]. Concerns about global warming have led to interest in biotechnological processes that might influence the potential removal of carbon dioxide from the atmosphere and production of biomaterials. Proteomic analysis has become important methods to establish coherences or correlations between biological, climatic or other effects and the composition of ecosystems or biological communities [5].

Some microorganisms are able to grow in limiting CO_2_ concentrations by employing a CO_2_-concentrating mechanism (CCM). Cyanobacteria and chemolithototrophic bacteria have ability to accumulate inorganic carbon in their cytoplasm. The CCM process takes place in polyhedral protein micro-compartments known as carboxysomes [6]. Carboxysomes contain the majority of enzymes mainly ribulose-1,5-bisphosphate carboxylase/oxygenase (RuBisCO), other carboxylating enzymes and carbonic anhydrase which facilitate the CO_2_ fixation [4], [7]–[9]. The organic biomass produced by photosynthetic microalgae and cyanobacteria can be transformed into biofuels, food additives, health-care products [10], [11].

Proteome analysis has become a powerful tool for investigating changes in prokaryotic protein expression [12]–[14]. Since 2-Dimensional (2D) gel electrophoresis analysis showed all bacterial soluble proteins expressed on gel at specific culture conditions, high throughput screening of these induced proteins is possible [15]. We have applied this approach to carbon dioxide sequestration in order to characterize the proteins induced by sodium bicarbonate/carbon dioxide. The proteome analysis has been performed upon several bacteria and cynobacteria including *Synechocystis* sp. PCC 6803 [16], *Synechocystis* 6803 [17]–[18] and *Riftia pachyptila* [19]. Carbon dioxide has been found to induce not only the expected CO_2_ sequestration related enzymes such as RuBisCO including various carboxylases and also other metabolic enzymes but also produces value added products, therefore, it is necessary to identify other metabolic processes in the CCM in bacteria by proteomic and metabolomic analysis [20]–[22]. In this study, chemolithotrophic bacteria was isolated from palaeoproterozoic metasediments to evaluate carbon dioxide sequestration mechanisms by proteomic analysis. In addition, carbonic anhydrase enzyme was partially purified which facilitates CO_2_ fixing enzyme RuBisCO for sequestration of carbon dioxide in the environment.

## Methods

No Specific permission was required for sampling of marble rock. The field studies did not involve endangered or protected species and was a suitable location for our study.

### Sampling site and microorganism

Samples were collected from marble rock of the palaeoproterozoic metasediments of the Aravali supergroup in the Umra area (27° 34′ N, 76° 38′ E), Rajasthan, India, for isolation of microorganisms. The upper portion of the rock was scrapped and dissolved in autoclaved distilled water (1∶10 w/v) which served as inoculums for enrichment of bacteria.

### Enrichment and culture condition of bacteria

A chemostat culture was set up in a 2-l glass vessel, effective volume 1 l, with culture condition as stirring at 150 rpm; temperature at 30°C; and pH 7.6 in the minimal salt medium (MSM). The composition of MSM (g/L) was: Na_2_HPO_4_, 7.8; KH_2_PO_4_, 6.8; MgSO_4_, 0.2 g; NaNO_3_, 0.085; ZnSO_4_.7H_2_O, 0.05; ZnCl_2_, 0.02; Ca(NO_3_)_2_.4H_2_O, 0.05 [23]. The supernatant containing microorganisms (50 ml) obtained from marble rock served as inoculums in the chemostat. The chemostat culture was run in presence of sodium bicarbonate (5 mM) initially, which increased gradually to 10, 20, 50, 100 and 150 mM, and growth of microorganisms were determined. After stabilization of growth of bacteria, the concentration of sodium bicarbonate was increased in the chemostat. Bacterial community from chemostat enriched in presence of sodium bicarbonate (5, 10, 20, 50, 100 and 150 mM) was inoculated in MSM in flasks for further enrichment. The culture medium was removed after 12, 24, 36, 48, 60 and 72 h for determining the growth pattern and carbonic anhydrase activity. The growth patterns of bacterial strains were taken as O.D. at 595 nm with a spectrophotometer (Cary, 100 Bio, Varian Co, Australia). One of the bacterial strains which survived in the sodium bicarbonate (150 mM) without losing its ability for production of carbonic anhydrase was cultured on LB-agar plate and selected for further study.

### Identification of bacteria by 16S rRNA method

Genomic DNA from the bacterial strain was isolated with the Genome DNA Kit (Qiagen Inc., USA) as described by the manufacturer. The 16S rRNA gene was amplified from genomic DNA by using PCR with universal primers 5′-GAGAGTTTGATCCTGGCTCAG–3′ (forward) and 5′-CTACGGCTACCTTGTTACGA-3′ (reverse) [24]. The amplified DNA was purified using Qiaquick PCR Purification Kit (Qiagen Inc., USA), adjusted to 200 ng/μl, cloned in the pDrive (Qiagen Inc., USA) and sequenced. Sequenced data was compared and analyzed with the existing database of Gene Bank, National Center for Biotechnology Information. A phylogenetic tree was drawn on the basis of the sequences. A bootstrap consensus tree (1,000 copies) was drawn by multiple sequence alignment with Neighbor-Joining method using software Mega, version 3.1 with different species of bacteria [25].

### Carbonic anhydrase enzyme assay and purification

Culture medium was centrifuged at 7500 rpm for 5 min, cell pellet was washed, suspended in sonication buffer (10 ml) containing Tris-HCl (50 mM, pH 6.5) buffer and lysozyme (0.2%) and sonicated for 10 min. After centrifugation at 12,000 rpm, the pellet was discarded and supernatant containing enzyme was stored. The protein concentration was determined by Bradford method with Bovine Serum Albumin as the standard. The carbonic anhydrase activity was measured in aqueous phase by using nitrophenyl esters [26]. The enzymatic reaction contained Tris-HCl buffer (50 mM, pH 7.5), 3 mM p-nitrophenyl acetate (p-NPA), and enzyme preparations (100 μL). One unit of enzyme activity was expressed as 1 μmol of p-nitrophenyl acetate hydrolyzed per minute.

Enzyme present in cell extract was precipitated by ammonium sulfate (30–75%) and the mixture was stirred for 2 h, and centrifuged at 15,000×*g* for 30 min. The ammonium sulphate fraction was dialyzed against Tris-HCl buffer (50 mM, pH 6.5) and applied to Sephadex G-100 gel filtration column. Fractions were collected and protein and carbonic anhydrase activity was determined. Partially purified enzyme of gel filtration was applied to the *p*-aminobenzenesulfonamide affinity column equilibrated with 25 mM Tris_HCl and 0.1 M Na_2_SO_4_ (pH 7.0) [27]. The affinity gel was washed with 25 mM Tris-HCl and 22 mM Na_2_SO_4_ (pH 7.0), and CA isozymes were eluted under different elution conditions [26]. Fractions were collected and the absorbance of fractions was measured at 280 nm. The carbonic anhydrase activities of fractions were measured. Selected fractions which gave carbonic anhydrase enzyme activity were analyzed by SDS-PAGE (12%) [28]. Proteins of known molecular mass were used as reference standards in comparison to carbonic anhydrase for molecular mass determination.

### RuBisCO Enzyme assay, purification and Western blot analysis of proteins

RuBisCO assay using spectrophotometric method was performed as described by Sharkey *et al*. [29]. RuBP-dependent oxidation of NADH was used to monitor activity of RuBisCO. The initial rate of oxidation of NADH in bacterium crude extract and protein fractions was determined by decrease in A_340_, using a ε340 of 6.220 μM^−1^ cm^−1^. Reactions were performed in 1.0-mL quartz cuvettes with a 1-cm light path. Activities were assayed at 4°C by adding 100 μL of crude protein to 500 μL of 200 mM sodium Tris-Cl buffer containing 10 mM MgCl_2_, 132 mM KHCO_3_, 10 mM DTT and 5.5 mg/ml ATP at pH 7.8 (Buffer A). 5 Unit (100 μL) of each of coupling enzymes glyceraldehyde-3-phosphate dehydrogenase (GPDH) and phosphoglycerate kinase (PGK), 2 mM NADH (100 μL) were added and mixed well. 2.5 mM D-Ribulose 1,5-diphosphate (RuBP) (200 μL) was added in reaction mixture, mixed quickly and effectively. Activity of RuBisCO was recorded using spectrophotometer and was expressed as the amount of enzyme that catalyzes the oxidation of 1 μmol of NADH per min. 4 μmoles of NADH are oxidized for each μmole of D-Ribulose 1,5-diphosphate utilized. One unit will convert 1.0 μmole of D-RuDP and CO_2_ to 2.0 μmoles of D-3-phosphoglycerate per minute at pH 7.8.

Bacterium cell extract was precipitated by ammonium sulphate with continuous stirring to achieve about 30–70% saturation for RuBisCO. The ammonium sulphate fraction was dialyzed against buffer A in a dialysis bag. The crude protein was loaded onto a DEAE cellulose column chromatography followed by a Superdex 200 column fast performance liquid chromatography (FPLC). The partially purified enzyme preparation was initially fractionated by DEAE cellulose ion exchange chromatography column (size: 1.5×20 cm). Dialysis membrane mediated desalted crude protein was applied to the top of the column, which was initially washed by Tris-HCl buffer (50 mM, pH 7.6), and subsequently with a linear gradient from 0 to 500 mM NaCl, at a constant flow rate of 0.5 ml/min. Fractions (5 mL each size) were collected and the absorbance of each fractions was measured at 280 nm. The fractions were tested for enzyme activity. Glass column (size 2×60 cm) with Superdex-200 was used for FPLC size exclusion chromatography. The column was washed extensively with buffer A. Active enzyme preparation of ion exchange column was loaded on the column. Flow rate of column was maintained at 0.5 ml/min. Absorbance of each fraction was measured at 280 nm, and fractions having higher absorbance, were checked for enzyme activity. Enzyme eluted from the Superdex 200 column was assessed for purity by SDS-PAGE while the molecular weight was confirmed using both high and low range molecular weight markers. In this method, acrylamide gel (12%) was used, and electrophoresis was performed. Gels were stained by Comassie brilliant blue (CBB) [28], [30]. Unstained Protein Molecular Weight Marker, a mixture of seven native proteins (14.4 kDa to 116 kDa) was used, in electrophoresis (SDS-PAGE).

In immunoblot analysis, crude protein, ammonium sulphate precipitated protein, DEAE purified protein and Superdex 200 purified proteins separated by SDS-PAGE (12%) were transferred to nitrocellulose membrane with Mini trans-blot electrophoretic transfer cell (GE Healthcare) according to directions provided by the manufacturer. After transferring, membrane was incubated in blocking solution containing 5% skimmed milk in 1X TBS for 12 h at 37°C in a slow rocker. This was followed by washing with 1X TBST twice for 20 min and a final rinse with 1X TBS. Membrane was then incubated for 4 h at 37°C with primary antibody [Anti-RuBisCO (plant) antibody produced in chicken] against rbcL (1∶500). This was followed by washing with 1X TBST twice for 20 min and a final rinse with 1X TBS. Membrane was then subjected to treatment with peroxidase conjugated goat anti-chicken IgG diluted to 1∶10000 (Secondary Antibody) for 45 min. This was again followed by washing with 1X TBST for 20 min followed by a final rinse with IX TBS. Horseradish peroxidase-labeled secondary antibodies and enhanced chemiluminescence (Amersham) were used for detection of the antibody-antigen conjugate. The antibody antigen complex was then detected using enhanced Chemiluminescence (ECL). For this, 10 ml of 100 mM Tris-Cl (pH 8.5) containing 50 μL of 250 mM luminol, 22 μL of 90 mM p-coumaric acid and 4 μL hydrogen peroxide was added into the membrane. The chemiluminesce was visualized with the help of image quant LAS 4000 imager (GE healthcare) and images were acquired with software.

### Two dimensional gel electrophoresis

For proteomic analysis, cell pellet was washed with distilled water, resuspended in lysis buffer (1 mL) containing 8 M urea, 4% (w/v) 3-[(3-Cholamidopropyl)dimethylammonio]-1-propanesulfonate hydrate (CHAPS), 2% pharmalytes and 1 mM phenyl methyl sulfonyl fluoride (PMSF) followed by sonication for 5 sec thrice. After centrifugation at 12,000 rpm, for 30 min, the pellet was discarded. Supernatant was used for determination of protein concentration by Bradford method using bovine serum albumin (Sigma Chemicals) as the standard. Cleanup kit was used for removal of salt according to the recommendations of the manufacturer (GE Healthcare Bio-Science). Thereafter, first dimension gel electrophoresis using 7 cm IEF (pH 3–10) strip followed by 2D gel electrophoresis (12% polyacrylamide) was performed.

Isoelectric focusing was performed by commercially available IPG-strips (pH 3–10, GE Healthcare Bio-Science). Samples were loaded by rehydration for 16 h in a solution containing 8 M urea, 1% (w/v) CHAPS, 20 mM DTT and 0.5% (v/v) ampholytes 3–10. The isoelectric focusing was performed with the Ettan IPGphor 3 IEF system (GE Healthcare Bio-Science) employing the following voltage profile: linear increase from 0 to 500 V for 500 Vh, 500 V for 2500 Vh, linear increase from 500 to 3500 V for 10 000 Vh, and a final phase of 3500 V for 35 000 Vh. After consecutive equilibration of the gels in solutions containing dithiothreitol (DTT) and iodoacetamide (IAA), the separation in the second dimension was performed in 12% polyacrylamide gels on the 2D gel electrophoresis system (GE Healthcare Bio-Science) as suggested by Görg *et al*. [31]. Gels were stained with CBB which was followed by destaining.

Gel images were captured using the Fluorchem (Alpha Innotech) densitometer at a resolution of 600×600 pixels and were analyzed with the PDQuest version 7.0.1 software (GE Healthcare Bio-Science). Spot detection and matching was performed using an automated function which was followed by manual designation of spots as landmarks for alignment of the gels. Spot intensities were normalized according to the mode “total of all valid spots” and analyses were performed using the quantitative and qualitative modes. The confidence threshold for up- and down-regulation of protein spots was set at two-fold below or above the spot intensity seen in the control.

### MALDI-TOF mass spectrometry and analysis of peptide sequences

Protein band visualized after Coomassie brilliant blue R staining in SDS-PAGE gel was subjected to MALDI-TOF/MS analysis (Brucker Daltonik (GmbH) [32]. Mass spectral data were analyzed by using data explorer software (Applied Biosystems, Foster City, CA). Acquired data were analyzed by comparison to in silico information contained in the NCBI databases using peptide mass fingerprinting (PMF). The 300 most intense peaks were searched against the NCBI taxonomy subset “all bacteria” (>753,000 sequences) at a mass tolerance of 50 to 100 ppm using mascot (http://www.matrixscience.com). Presence of the metabolic enzymes was confirmed by submission of detected fragment ions to the NCBI database.

## Results and Discussion

### Enrichment and identification of carbon dioxide concentrating bacteria

The microorganisms isolated from soil and sediment of marble mining site was enriched in the chemostat in presence of MSM and increasing concentration of sodium bicarbonate as sole carbon source. One bacterial isolate which survived in MSM containing sodium bicarbonate (150 mM) was cultured on LB-agar plate. The isolate was identified by 16S rDNA analysis as *Serratia* sp. showing 99% homology in the BLAST nucleotide search program of the National Center for Biotechnology Information. The sequence after matching by phylogenetic tree showed relatedness with *Serratia* sp. (Gene Bank Accession No. JF276275). *Serratia* sp. ISTD04 is a motile, Gram-negative, rod-shaped facultative anaerobe.

### Enzyme assay and partial purification of carbonic anhydrase

**Figure 1:**
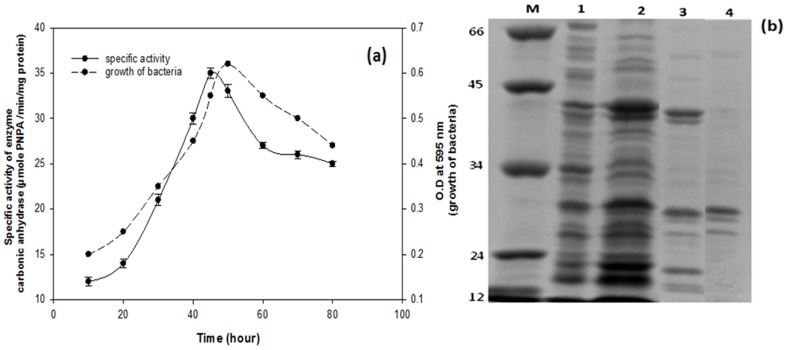
Growth of *Serratia* sp.ISTD04 isolated from bacterial community enriched in sodium bicarbonate (150 mM) in the chemostat further grew in minimal salt medium with sodium bicarbonate as sole carbon source. At right y2-axis, dotted line represents bacterial Growth at O.D. 595 nm with respect to time in hours and at left y1-axis, bold line represents specific activity of crude enzyme (carbonic anhydrase) with respect to time in hour and at x –axis (a). SDS-Polyacrylamide gel electrophoresis of carbonic anhydrase of *Serratia* sp.ISTD04. In figure, lane M is marker, lane 1 is of crude protein of bacterium, lane 2 is precipitated protein after ammonium sulphate precipitation, lane 3 is protein of Sephadex G 100 gel filtration chromatography and lane 4 is protein obtained after affinity chromatography (b).

The growth of bacterium, *Serratia* sp. ISTD04, and production of carbonic anhydrase were assayed. Results of the study indicated that the enzymatic activity and growth of bacteria increased after 6 h and reached to maximum at 48 h, and then declined (Fig. 1a). The carbonic anhydrase (CA) enzyme of *Serratia* sp. ISTD04 was purified by ammonium sulphate precipitation, gel filtration chromatography and affinity chromatography. The crude soluble extract subjected to ammonium sulfate precipitation (30–75%) exhibited specific activity 29.3 U/mg, 2.25% fold purification and yield was 80.71%. Partially purified protein was fractionated by Sephadex G-100 gel filtration to remove other proteins on the basis of molecular size. Four fractions were obtained in which one of the fractions; molecular weight 29 kDa, determined by known molecular weight markers had activity of carbonic anhydrase (Fig. 1b). Results indicated specific enzyme activity of 81.2 U/mg, 6.24% fold purification and yield was 69.16. Partially purified enzyme of Sephadex G-100 was further purified by affinity column chromatography. Results indicated specific enzyme activity 704.91 U/mg, 54.22% fold purification and yield was 57.23. In this study, carbonic anhydrase showed higher activity of enzyme responsible for efficient CO_2_ sequestration as it is a supporting enzyme for the activity of RuBisCO. This enzyme augments and enhances carbon fixation activity. Its primary role is to facilitate the presence of enough CO_2_ molecules for RuBisCO through reversible conversion of CO_2_ into bicarbonate ion as CO_2_ molecules escape easily through the cell membrane as opposed to bicarbonates. RuBisCO needs high CO_2_ concentration to exhibit carboxylase activity and is often out competed by oxygenase activity. The CA enzyme is thought to dehydrate abundant cytosolic bicarbonate and provide RuBisCO to fix carbon dioxide within the carboxysome. The concentrations of CO_2_ to permit its efficient fixation to ribulose 1, 5-bisphosphate and hence availability of CO_2_ per minute towards the active site of the RuBisCO may enhance and in this way fixation of CO_2_ would be enhanced [33]. Some chemolithotropic bacteria and most of cyanobacteria have proper channels for transport of dissolved inorganic carbon (CO_2_, HCO_3_^−^, and CO_3_^2−^). Bacteria accumulate HCO_3_^−^ in cytoplasm and trigger higher CA activity by enhancing the bicarbonate ion concentration or gaseous CO_2_ concentration for conversion of HCO_3_^−^ to CO_2_. In this way, bacteria acclimates itself to sequester more CO_2_. So activity of carbonic anhydrase is important factor for CO_2_ sequestration by RuBisCO [33].

### Enzyme assay, purification and western blot analysis of RuBisCO

**Figure 2:**
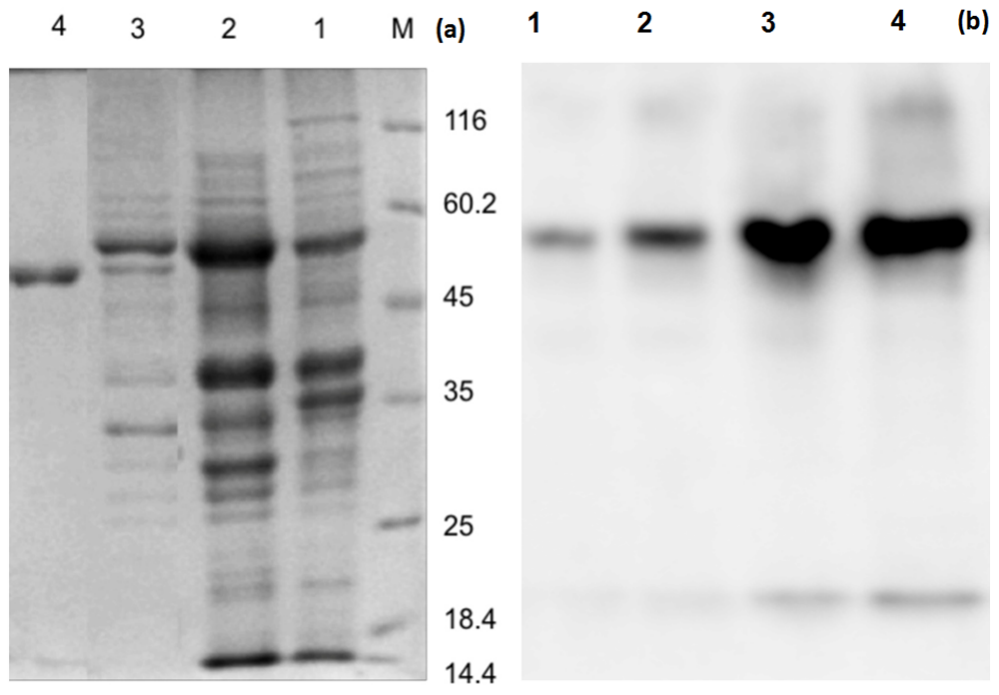
SDS–PAGE for purification of RuBisCO. In Lane M, protein molecular weight markers of different molecular weight (14.4, 25, 35, 45, 60.2 and 116kDa); in lane 1, 5.6 μg crude lysate of induced bacteria with sodium bicarbonate; in lane 3, 4 μg of protein from DEAE; in lane 4; 0.8 μg of protein from the FPLC superdex 200 (a). Western blot with antibodies against the rbcL. 1, crude protein from *Serratia* sp.ISTD04; 2, protein from (NH_4_)_2_SO_4_ ppt 20-75% cut; 3, protein from DEAE; 4, purified RuBisCO by FPLC Superdex 200 (b).

RuBisCO enzyme was assayed, isolated and purified to homogeneity at 4°C from *Serratia* sp. ISTD04 cell extracts grown on MSM with sodium biocarbonate (150 mM). The RuBisCO activity in crude enzyme solution was 8.94 nanomole CO_2_/mg protein/min. The RuBisCO enzyme was purified by ammonium sulphate precipitation, ion exchange and gel filtration chromatography. The molecular weight of the enzyme was estimated to be 5,50,000 Dalton by FPLC gel filtration. The enzyme consists of two subunits, whose molecular weights were 56 and 14 kD as determined by SDS-PAGE, respectively (Fig. 2a). These results indicated that the enzyme consists of eight large subunits and eight small subunits. The Western blot analysis of the crude protein sample and purified sample showed strong band pattern at molecular weight around 56 kDa regions (Fig. 2b). This result confirmed the presence of RuBisCO in purified protein of *Serratia* sp. ISTD04. RuBisCO antibodies had approximate apparent molecular masses of 56 kDa. These probable RuBisCO large-subunit polypeptides were more prominent in extracts in cells of *Serratia* sp. ISTD04 (Fig. 2b). RuBisCO activity of *Serratia* sp.ISTD04 was low as compared to those in higher plants showing the lower end value of 50 nanomole CO_2_/mg protein/min in *Symphytum* sp. and it was much lower than 540–750 nanomole CO_2_/mg protein/min in *Nicotiana tabacum* and 1700–2100 nanomole CO_2_/mg protein/min in spinach [34]. Hilditch *et al.* (1991) reported the activities were 15.5, 9.4, 6.1, 52.7, 24.2 and 18.8 nanomole CO_2_/mg protein/min in *Enteromorpha prolifera, Ulva lactuca*, *Chonrrus crispus, Corallina officinalis, Dumontia incrassate and porphyra umbilicalis*, respectively [35].

### 2D gel electrophoresis and MALDI-TOF/MS

Whole cell soluble protein of *Serratia* sp. ISTD04 grown under autotrophic and heterotrophic conditions was extracted. The proteins were resolved on broad-range (pH 3–10) by IPG strips. The pI region 3–10 represents the major part of the cytosolic proteome. Upon assigning apparent pI values on broad-range 2D gel electrophoresis image using the PDQuest program, it was observed that more than 75% of the soluble proteins fall within a range of pH 4.5–7.0 under both autotrophic and heterotrophic metabolic modes. IEF and SDS-PAGE were performed in triplicate for samples from both autotrophic and heterotrophic conditions so as to get differential protein spots resolved on gel surface of 2D gel electrophoresis for comparative analysis. Depending upon the amount of protein sample applied to the gels and the spot detection parameters, 953–970 protein spots were observed on the pH 4.5–7.0. Quantitative comparison of more than 200 spots was done using PDQuest to estimate fold changes of the protein spots.

**Figure 3:**
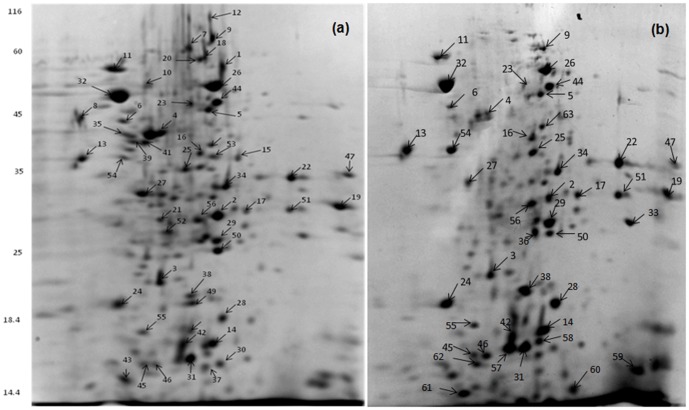
Two dimensional PAGE gel images of soluble proteins from heterotrophically grown *Serratia* sp. cells after staining Coomassie brilliant blue. Proteins were separated in the first dimension on an IPG strip pH–10.0 and in the second dimension on a 12% SDS PAGE-gel (a). 2DE-PAGE gel images of soluble proteins from autotrophically grown *Serratia* sp. ISTD04 cells after staining with Coomassie brilliant blue. Proteins were separated in the first dimension on an IPG strip pH 3.0–10.0 and in the second dimension on a 12% SDS PAGE-gel (b).

Out of the 953 protein spots reproducibly visible and assigned on different gels, only 63 spots were identified by using MALDI-TOF/MS followed by database searching (Table 1). This covered most of the spots that consistently showed differential expression under autotrophy compared to heterotrophy. Out of 63 protein spots, 48 spots were significantly up-regulated in the autotrophically grown cells; seven enzymes were found and showed its utilization in autotrophic carbon fixation pathways. Fifteen enzymes were utilized in Fatty acid metabolism and four of them were those enzymes which were utilized in hydrocarbon synthesis. The mass spectrometric analysis of four well-resolved spots (spots 13, 33, 48 and 63) showed the presence of peptides unique to two different proteins, and hence it could be concluded that these spots contain two overlapping proteins. This study also showed the presence of four proteins in two or more spots on the 2D gel electrophoresis indicating different isoforms of the proteins (Fig. 3a, 3b).

**Table 1:** Proteins identified from the autotrophic and heterotrophic bacterial cells, *Serratia* sp.ISTD04, by MALDI-TOF/MS.

Spot no	Protein name	ThrpI/mass(KDa)	ExppI/mass(KDa)	Sequence coverage	MASCOT score
**CO_2_ fixing Enzyme**					
1	Rubisco Large subunit	7.8/55.5	7.6/53	21.1%	45
2	Carbonic anhydrase	6.5/29	7.5/29	29.6%	72
3	Transketolase	9.4/17.6	6.7/22	14.5	45
4	Aldolase	5.9/38.49	6.5/38	12%	36
5	Fructose-1,6-biphosphatase	7.8/45.14	7.8/44	21%	27
6	Glyceraldehyde-3 phosphate dehydrogenase	5.0/44.7	4.9/38	42.3%	51
7	PEPCK	6.0/66.8	6.8/60	27.9%	38
**TCA cycle Enzyme**					
8	Succinyl-CoA synthase	5.0/44.8	5/44	30.1%	47
9	Fumaratereductase, Flavoprotein	5.1/66.8	5/67	14.8%	37
10	NAD dependent malic enzyme	5.3/55	5.3/55	13%	43
11	Pyruvate carboxylase	6.7/56	6.7/56	17%	67
12	2-oxoglutarate dehydrogenase	4.7/109.1	4.7/100	23%	57
13	Citrate Synthase	4.8/48.54	4.2/40	25%	78
14	Aconitase	7.8/18.14	7.8/17.1	24%	67
15	Succinate dehydrogenase	9.4/40.1	8.1/39	14.5%	81
16	Malate dehydrogenase	5.9/38.49	6.6/40	32%	106
**Fatty acid Metabolism Enzyme**					
17	Fatty acid synthesis plsx protein	7.7/28	7.3/32	22.1%	37
18	Cyclopropane fatty acid synthase	7.7/71.9	7.2/71	23.3	39
19	Acetyl CoA C- acyltranferase	6.8/46.3	9.2/32	29.1%	47
20	2,4-dienoyl-CoA reductase	9.2/73.7	7.4/58	8.4%	45
21	Enoyl –CoA hydratase/isomerase	5.5/29.2	5.5/30	19.8%	41
22	Lipoic acid synthatase	9.4/25.3	8.7/33	51.1%	36
23	Acyl coAthiolase	6.8/46.2	6.8/46	34.4%	73
24	Acetyl transferase GNAT	5.2/20.25	5.2/20	21%	54
25	Acetyl CoA carboxylase	5.7/34.4	6.7/34.4	16%	46
26	Acyl CoA dehydrogenase	6.8/71.92	6.8/50	29.6%	47
27	Fatty acid desaturase type I	6.2/35.99	5.8/32	28%	98
28	3-Oxoacyl-(Acyl-cerrier-Protein) reductase	6.1/27.9	8/20	31%	81
29	3-Oxoacyl-(Acyl-cerrier-Protein) Synthase III	6.4/37.22	7.5/31	27%	33
30	Holo (Acyl carrier protein) synthase	9.9/13.5	7.8/16	19%	50
31	Triacylglycerol lipase superfamily	6.3/40.9	6.4/19	12%	37
**Amino acid Metabolism**					
32	Propionyl-CoA carboxylase	5/73.49	4.3/53	35.6%	43
34	Shikimate-5-dehydrogenase	7.6/31.1	7.6/34	31%	41
35	Glutamate synthase	5/48.32	4.8/41	21%	52
36	4-Hydroxy phenyl pyruvate dioxygenase	5.4/28	5.9/27	27.1%	111
37	Branched-chain alpha-keto acid dehydrogenase	7.9/7.6	7.3/15	27.5%	91
38	3-Hydroxy isobutyrate dehydrogenase	6.3/20.0	6.1/20	18%	45
39	Diaminopimelateepimerase	5.6/49.78	5.6/41	29%	56
40	3-phosphoshikimate 1-carboxyvinyltransferase	5.7/46.9	7.5/38.2	18%	39
**Translation**					
41	Elongation factor Tu	4.5/43.3	6.5/41	34.1%	83
**Ribosomal Protein**					
42	50S ribosomal protein L18	15.5	6.1/16	15%	44
43	30S ribosomal protein S14	6.37/18	5.7/15.2	27%	42
44	30S ribosomal subunit protein S1	7.9/61.2	7.8/47	24.6%	87
45	50S ribosomal protein L23	6.8/11.3	6.2/15.6	31.4%	40
46	50S ribosomal protein L24	6.3/10.7	6.4/15.6	21.8%	25
**Nucleotide biosynthesis**					
47	Dihydrorotate dehydrogenase	9.4/39.08	9.4/31.8	31%	92
48	Inosine-5-monophateDehydrogenase	6.7/52.2	6.7/52.2	27%	57
49	Phosphoribosylaminoimidazolecarboxylase catalytic subunit	6.5/17.8	7/20	24.6%	89
**Alkane biosynthesis**					
50	Aldehyde dehydrogenase	6.0/52.8	7.6/26	27%	69
51	Short chain dehydrogenase	9.0/27.35	9/30	24.6%	72
52	Short chain Alcohol hydrogenase	5.2/27.3	5.8/27.3	30.4%	40
53	Geranylegernyle synthase	7.5/35.7	7.3/35.7	24.8%	29
**Vitamin synthesis**					
54	Thiamine biosynthesis	4.9/37.7	5.3/37.7	29%	38
55	Riboflavin synthase	4.9/18.7	6.1/18.1	18%	40
**Glycolysis**					
56	Phosphoglyceratemutase	5.2/28.8	7.1/28.8	31%	28.8
57	Fructose 1,6 bisphosphatase II	5.2/22	6.3/18	28%	98
58	D-3-phosphoglycerateDehydrogenase	5.9/57.9	6.9/18.2	27.1%	57.9
**Other protein**					
59	Hypothetical protein	7.5/21	7.3/17.3	27%	98
60	Hypothetical protein	5.5/18	5.5/14.6	26%	123
61	Hypothetical protein	5.8/17	5.5/14.6	23%	45
62	Hypothetical protein	6.2/20	5.8/17.3	19%	52
63	Rubisco transcriptional regulator	7.5/36	7.3/35.8	27%	42

The present study showed the expression profiles of different proteins that can be visualized from the 2D gel electrophoresis. The protein spots identified in the present study indicated RuBisCO large (spot 1), carbonic anhydrase (spot 2), propionyl-CoA carboxylase (spot 32), acyl-CoA dehydrogenase (spot 26), aldolase (spot 4), 30S ribosomal subunit protein S1(spot 44), 50S ribosomal protein L18 (spot 42), lipoic acid synthatase (spot 22), acetyl transferase GNAT (spot 24), D-3-phosphoglycerate dehydrogenase (spot 58), elongation factor Tu (spot 41), and fructose-1,6-biphosphatase (spot 5), showed a high predicted expression. The enzymes include malate dehydrogenase (spots 16), protein synthesis elongation factor Tu (spots 41), acyl-CoA dehydrogenase (spots 26) and acetyl-CoA carboxylase (spots 25) were also detected. Unresolved complexes were visible in all gels within a mass range of 14.4–16 kDa (pH 9.1–9.9) and 15.2–16.1 (6.3–6.6) (Fig. 3a and 3b). On a functional basis, the identified proteins can be described to various cellular processes such as carbohydrate metabolism, fatty acid metabolism, protein synthesis, nucleotide and amino acid metabolism, and hydrocarbon biosynthesis.

### Proteins involved in carbon metabolism

Transition from autotrophy to heterotrophy brought about changes in the expression levels of several enzymes involved in central carbon metabolism (Table 1). RuBisCO large subunit (RbcL) (spot 1) was greatly reduced under heterotrophy. Result of the MALDI-TOF and MS/MS indicated presence of large fragment of RuBisCO and RuBisCO transcriptional regulator (spot 63). Other proteins involved in the CO_2_ concentrating mechanism whose expression was reduced during heterotrophy includes carbonic anhydrase (spot 2), phosphoenol pyruvate carboxykinase PEPCK (spot 7), pyruvate carboxylase (spot 11), acetyl CoA carboxylase (spot 25) and propionyl-CoA carboxylase (spot 32). In hetrotrophic condition, low expression of calvin cycle enzyme, carboxylases enzyme and reduced chemosynthetic capacity explains the proteomic expression level changes due to environment and stress conditions. In 3-hydroxypropionate pathways, CO_2_ fixing enzymes were acetyl-CoA carboxylase and propionyl-CoA carboxylase. This pathway was discovered in the green non sulfur bacterium *Chloroflexus*. Here, CO_2_ fixation starts with the carboxylation of acetyl-CoA; the CO_2_ acceptor was regenerated in a cyclic process, with 3-hydroxypropionate and malyl-CoA as characteristic intermediates [36]. However, fructose-1,6-biphosphatase (spot 5), an enzyme involved in calvin cycle, did not show any change in the expression level under both conditions. Its presence in both conditions could be explained by the fact that the enzyme also participates in glycolysis which was prevalent in both autotrophy and heterotrophy.

Heterotrophic growth of *Serratia* sp. ISTD04 showed the effective utilization of exogenously supplied glucose as energy and carbon sources. In chemolithotrophic bacteria, the pentose phosphate pathway (OPP) had been suggested to act as the sole route of hexose dissimilation [37]. However, glucose 6-phosphate dehydrogenase (G6PDH) was over expressed in heterotrophy condition. It is the key enzyme controlling carbon flow into the OPP pathway in bacteria. A facultative photo- and chemoheterotroph dissimilates glucose with formation of CO_2_ as the only major product. A substantial fraction of the glucose consumed is assimilated and stored as polyglucose (probably glycogen). The oxidation of glucose proceeds through the pentose phosphate pathway. The first enzyme of this pathway, glucose-6-phosphate dehydrogenase, is partly inducible. In addition, the rate of glucose oxidation is controlled, at the level of glucose-6-phosphate dehydrogenase function, by the intracellular level of an intermediate of the Calvin cycle, ribulose-1,5-diphosphate, which is a specific allosteric inhibitor of this enzyme [38]. The expression levels of transaldolase (spot 3) and transketolase (spot 4), the two enzymes in the regenerative steps of the OPP pathway increased by 1.2- and 2.4-fold, respectively, under autotrophy. The OPP pathway and pentose sugars required for cellular biosynthesis, therefore, seems active and crucial during heterotrophic growth.

NAD dependent malic enzyme was expressed in only autotrophic condition and was completely absent in heterotrophic condition. In some C4 plant species, a mitochondrial NAD-dependent malic enzyme (EC 1.1.1.39) (NAD-ME) catalyzes the decarboxylation of 4 carbon malate in the bundle sheath cells, releasing CO_2_ for the Calvin cycle of photosynthesis [39]. One of the most significant proteomic changes observed in the present study was the enhanced expression level of citrate synthase (spot 13), malate dehydrogenase (spot 16) and aconitase (spot 17) under heterotrophy. Similarly, in the present study, all these enzyme activity showed several folds increase consistent with the protein levels under heterotrophy (Table 1). However, the functional significance of this increased expression under heterotrophy had not been explained in *Serratia* sp. ISTD04. This is particularly because malate dehydrogenase (MDH), as described in higher plants, is involved in a variety of biochemical pathways such as the TCA cycle, anaplerotic pathways, and malate valve and these pathways are completely or partly confined to cell organelles [40]. In bacteria and cynobacteria, however, intracellular compartmentalization is limited, allowing by passing of different pathways and their reaction intermediates. Other proteins involved in TCA cycle such as succinyl-CoA synthase (spot 8), fumarate reductase (spot 9), 2-oxoglutarate dehydrogenase (spot 12), and succinate dehydrogenase (spot 15) showed a significant expression during autotrophic growth. In the reverse TCA cycle, CO_2_ and water are taken to make carbon compounds. This process is used by some bacteria to synthesise carbon compounds, sometimes using hydrogen, sulfide, or thiosulfate as electron donors [41]. An incomplete TCA cycle has been found in a surprisingly large number of bacterial pathogens including *Helicobacter pylori, Haemophilus influenzae* and *Streptococcus mutans* [42]–[44]. The primary role of the TCA cycle is to provide NADH which is used by bacterial cells for ATP synthesis via the electron transport chain (ETC). However, the TCA cycle also plays a key role in the synthesis of intermediates for anabolic pathways; specifically 2-ketoglutarate, oxaloacetate and succinyl-CoA which are starting points for the synthesis of glutamate, aspartate and porphyrin respectively. Bacteria that harbor an incomplete TCA cycle retain the ability to generate 2-ketoglutarate, oxaloacetate and succinyl-CoA from pyruvate.

### Proteins involved in amino acid metabolism

The data of study showed increased expression levels of propionyl-CoA carboxylase (spot 32), shikimate-5-dehydrogenase (spot 34), glutamate synthase (spot 35), branched-chain alpha-keto acid dehydrogenase (BCKDC) (spot 37), diaminopimelate epimerase (spot 39) and 3-phosphoshikimate 1-carboxyvinyltransferase (spot 40) involved in amino acid metabolism, refer to both active biosynthesis and degradation of different amino acids under autotrophy. Shikimate-5-dehydrogenase (spot 34) and 3-phosphoshikimate 1-carboxyvinyltransferase (spot 40) are both involved in aromatic amino acid synthesis in bacteria. These pathways are found in bacteria, plants, fungi, algae, and parasites and are responsible for the biosynthesis of aromatic amino acids (phenylalanine, tyrosine, and tryptophan) from the metabolism of carbohydrates [45]. Glutamate synthase (spot 35) participates in glutamate and nitrogen metabolism. It has 5 cofactors: FAD, Iron, FMN, Sulfur and Iron-sulfur. In animal tissue, branched-chain α-ketoacid dehydrogenase complex (BCKDC) catalyzes an irreversible step in the catabolism of branched-chain amino acids known as L-isoleucine, L-valine, and L-leucine [46]. In bacteria, this enzyme participates in the synthesis of branched, long chain fatty acids [47]. In plants, this enzyme is involved in the synthesis of branched, long chain hydrocarbons [48]. 3-Phosphoshikimate 1-carboxyvinyl transferase catalyses the sixth step in the biosynthesis from chorismate of the aromatic amino acids (the shikimate pathway) in bacteria (gene aroA). In plants and fungi (where it is part of a multi functional enzyme) it catalyses five consecutive steps in the shikimate pathway [49]. Decreased expression levels of 4-hydroxy phenyl pyruvate dioxygenase (spot 36) and 3-hydroxy isobutyrate dehydrogenase (spot 38) involved in amino acid metabolism shows degradation of tyrosine, valine, leucine and isoleucine amino acids respectively under autotrophy.

### Proteins involved in fatty acid metabolism

Fatty acid biosynthesis and degradation requires carrier proteins and enzymes involved in the addition and subtraction reactions of acetate unit to a hydrocarbon chain. Acetyl-CoA carboxylases (ACCs) are the key enzymes and mediate a carboxylation reaction to produce malonyl-CoA from acetyl-CoA. The most important function of ACC is to provide the malonyl-CoA substrate for the biosynthesis of fatty acids [50]. Other key enzyme is fatty acid synthase consisting of six enzymatic activities and it is responsible for the reactions of adding acetate unit to a growing fatty acid chain. In this study, ACCs were up-regulated in the autotrophic medium in contrast to the glucose medium (Table 1). This finding is quite similar to the previously reported results as ACC was up-regulated in the acetate medium to generate polyhydroxyalcanoates (PHAs) in *Ralstonia eutropha* [51].

Increased expression of acetyl CoA C-acyltranferase (spot 19), 2,4-dienoyl-CoA reductase (spot 20), acyl CoA dehydrogenase (spot 26), fatty acid desaturase type I (spot 27) and triacylglycerol lipase superfamily (spot 31) involved in beta oxidation of fatty acid metabolism, under autotrophic condition was observed. Acetyl CoA C-acyltransferase participates in the final step of β oxidation of fatty acid to form acetyl CoA molecule and an acyl CoA molecule. 2,4 dienoyl-CoA reductase is an accessory enzyme that participates in the beta oxidation and metabolism of polyunsaturated fatty enoyl-CoA esters. Specifically, it catalyzes the reduction of 2,4 dienoyl-CoA thioesters of varying length by NADPH cofactor to 3-trans-enoyl-CoA of equivalent length. 2,4 Dienoyl-CoA reductase (DECR) from *Escherichia Coli* shares very similar kinetic properties to that of eukaryotes, but differs significantly in both structure and mechanism. In addition to NADPH, *E. Coli* DECR requires a set of FAD, FMN and iron-sulfur cluster molecules to complete the electron transfer [52]. Acyl-CoA dehydrogenase catalyzes the initial step in each cycle of fatty acid β-oxidation. Their action results in the introduction of trans double-bond between C2 and C3 of the acyl-CoA thioester substrate [53]. For activity of this enzyme, FAD is a required as a co-factor to bind its appropriate substrate. Fatty acid desaturases type1 are enzymes that catalyze the insertion of a double bond at the delta position of fatty acid, in autotrophic growth condition of bacteria. Thus, it can be inferred that more β oxidation of fatty acid occurs under autotrophic condition in comparison to heterotrophic condition. In this experiment, various fatty acid biosynthesizing proteins involved in the lipid production were over-expressed in heterotrophic condition and they included 3-oxoacyl-(acyl-carrier protein) synthase III, enoyl-(acyl-carrier- protein) reductase and fatty acid synthesis plsx protein. 3-Oxoacyl-[acyl carrier protein (ACP)] synthase III catalyses the first condensation step within the FAS II pathway, using acetyl-CoA as the primer and malonyl-ACP as the acceptor. The oxoacyl-ACP formed by this reaction subsequently enters the elongation cycle, where the acyl chain is progressively lengthened by the combined activities of several enzymes. 3-Oxoacyl-(acyl-carrier-protein) reductase participates in fatty acid biosynthesis and polyunsaturated fatty acid biosynthesis. The plsX gene is part of the bacterial fab gene cluster which encodes several key fatty acid biosynthetic enzymes. The plsX gene encodes a poorly understood enzyme of phospholipid metabolism [54], [55]. In our study, enoyl-CoA hydratase/isomerase was found in both autotrophic and heterotrophic medium and they were not found to express differentially. It was observed that in lipid production, heterotrophic growth of bacteria is more significant than autotrophic.

### Proteins involved in nucleotide metabolism

Results of the study indicated a sharp up-regulation of nucleotide biosynthetic proteins such as dihydroorotate dehydrogenase (spot 47), inosine-5′-monophosphate dehydrogenase (spot 48), and phosphoribosyl aminoimidazole carboxylase (spot 49), under autotrophic growth condition. These proteins are involved in purine and pyrimidine biosynthesis, as well as synthesis of nucleoside triphosphates. Dihydroorotate dehydrogenase is an enzyme that catalyzes the fourth step in the de novo biosynthesis of pyrimidine. It converts dihydroorotate to orotate. In bacteria (gene pyrD), it is located on the inner side of the cytosolic membrane. In some yeasts, such as in *Saccharomyces cerevisiae* (gene URA1), it is a cytosolic protein, whereas, in other eukaryotes, it is found in the mitochondria [56]. Inosine-5′-monophosphate dehydrogenase (IMPDH) is an essential cytoplasmic purine metabolic enzyme that catalyzes the NAD-dependent oxidation of inosine monophosphate (IMP) to xanthosine monophosphate (XMP), the first and rate-limiting step towards the synthesis of guanosine triphosphate (GTP) from IMP. IMPDH has an essential role in providing precursors for DNA and RNA bioysynthesis [57]. Phosphoribosyl aminoimidazole carboxylase is an enzyme involved in nucleotide biosynthesis and in particular in purine biosynthesis

### Ribosomal proteins

Proteins from the large and small ribosomal subunits (50S/L18 (Spot 42), 50S/L23 (Spot 45), 50S/L24 (Spot 46) and 30S/S1 (Spot 44)) were down-regulated under autotrophic condition. Ribosomal protein 30S/S14 (Spot 43) was up-regulated, as observed by visual inspection, but they were not easily quantified because of poor spot resolution. During the hetrotrophic growth condition, the cell density increased linearly and then gradually plateaued with a decrease in the growth rate. The number of ribosomes per cell (ribosome density) is growth rate controlled and decreases as the growth rate declines. Therefore, a decrease in ribosomal protein levels could be anticipated and observed.

## Conclusions

This study provides insight into the dynamics of the carbon concentrating mechanism by bacterium *Serratia* sp. ISTD04 isolated from marble rock and enriched in the chemostat in presence of sodium bicarbonate as sole carbon source. The production of carbonic anhydrase enzymes and RuBisCO by bacterium indicated sequestering potency of carbon dioxide. Protein purification and Western blot analysis substantiated the presence and role of RuBisCO in bacterium. The present study is the first proteomic attempt where 2D gel electrophoresis coupled with MALDI-TOF/MS to characterize the differential expression of proteins under autotrophic and heterotrophic conditions in *Serratia* sp. ISTD04 has been done. Qualitative and quantitative comparison of several hundreds of protein spots was made and it was found that proteins like RuBisCO, carbonic anhydrase, PEPCK and other carboxylase enzyme were highly over expressed under autotrophic condition. However, under heterotrophic condition, over expression of proteins like fatty acid synthesis plsx protein, 3-oxoacyl-(acyl carrier protein) reductase, 3-oxoacyl-(acyl carrier protein) synthase III etc was observed which is used in lipid synthesis. Thus, it can be concluded that *Serratia* sp. ISTD104 is a potent strain for CO_2_ sequestration.
